# Better olfaction was associated with lower healthcare expenditure among physically independent Japanese community-dwelling older adults: the Yanai study

**DOI:** 10.3389/fragi.2025.1592838

**Published:** 2025-08-07

**Authors:** Yujiro Kose, Yoichi Hatamoto, Rie Tomiga-Takae, Yukari Kimuro, Ryo Aoyagi, Hikaru Kawasaki, Takaaki Komiyama, Mamiko Ichikawa, Katsutoyo Fujiyama, Yoshiro Murata, Masahiro Ikenaga, Yasuki Higaki

**Affiliations:** ^1^ Department of Sports and Life Science, National Institute of Fitness and Sports in Kanoya, Kanoya, Japan; ^2^ Sports Innovation Organization, National Institute of Fitness and Sports in Kanoya, Kanoya, Japan; ^3^ Fukuoka University Institute for Physical Activity, Fukuoka University, Fukuoka, Japan; ^4^ Department of Nutrition and Metabolism, National Institute of Health and Nutrition, National Institutes of Biomedical Innovation, Health, and Nutrition, Osaka, Japan; ^5^ Faculty of Sports and Health Science, Fukuoka University, Fukuoka, Japan; ^6^ Department of Nursing, Nishikyushu University, Saga, Japan; ^7^ Graduate School of Sports and Health Science, Fukuoka University, Fukuoka, Japan; ^8^ Center for Education in Liberal Arts and Sciences, Osaka University, Osaka, Japan; ^9^ Department of Sport and Medical Science, Teikyo University, Tokyo, Japan; ^10^ Yanai city hall, Yamaguchi, Japan; ^11^ Emu Kankyo Design System Co., Ltd., Fukuoka, Japan; ^12^ Faculty of Engineering, Nishinippon Institute of Technology, Fukuoka, Japan

**Keywords:** olfactory dysfunction, odor identification, orthopedics, Odor Stick Identification Test for Japanese, hyposmia

## Abstract

**Objective:**

We aimed to determine whether olfaction is associated with healthcare expenditure among physically independent, community-dwelling older adults.

**Methods:**

This cross-sectional study included 162 community-dwelling older adults (mean age 70.2 ± 5.4 years; 48 men and 114 women) from the 2015 Yanai Study. Of these, we followed 71 participants (mean age 70.0 ± 5.7 years; 26 men and 45 women) in a longitudinal pilot study over 1.5 years, which measured healthcare expenditure from May 2015 to October 2016. Olfaction was examined using the Odor Stick Identification Test for Japanese people.

**Results:**

The cross-sectional analysis showed that better overall olfaction was significantly associated with lower orthopedic expenditure at baseline when adjusted for covariates (*f* = 2.19; *p* = 0.115; *p* for linear trend = 0.048). This association remained significant in the model adjusted for final covariates (*f* = 2.30; *p* = 0.105; *p* for linear trend = 0.034). Better olfaction scores (≥8) were associated with lower orthopedic expenditure in the final covariate model [odds ratio (OR): 0.780; 95% confidence interval (CI): 0.612–0.994; *p* = 0.045]. The longitudinal analysis showed that a baseline olfaction score ≥8 was associated with lower total healthcare expenditure, outpatient visit expenditure, and internal medicine expenditure, independent of covariates over 1.5 years, in the model adjusted for final covariates (group × time interaction: *f* = 4.05, *p* = 0.021; *f* = 4.34, *p* = 0.016; and *f* = 6.20, *p* = 0.005).

**Conclusion:**

Both the cross-sectional and longitudinal analyses suggested that better olfaction ability is associated with lower healthcare expenditure. These results may help increase awareness of olfactory dysfunction at an earlier stage among physically independent, community-dwelling older adults.

## Introduction

The olfactory system is responsible for olfaction (sense of smell), and olfactory system dysfunction is linked to impaired early health outcomes in community-dwelling older people (Papazian and Pinto, 2021). Previous cross-sectional studies involving community-dwelling older adults have reported that olfactory dysfunction is associated with poor physical performance in this population (Tian et al., 2017; Kose et al., 2022; Yuan et al., 2024) and adults aged ≥40 years (Ramirez-Velez et al., 2021). Another study showed that olfactory impairment was closely associated with sarcopenia and frailty in community-dwelling older adults (Harita et al., 2019). Olfactory dysfunction was also associated with cognitive function (Delgado-Lima et al., 2023) and brain atrophy (Kose et al., 2021). In addition, longitudinal studies showed that olfactory function may be used to predict mortality (Pinto et al., 2014) and dementia (Knight et al., 2023). A previous study reported that poor olfaction predicted a faster decline in physical functioning (Yuan et al., 2021). Among community-dwelling older adults, including both cognitively impaired and cognitively normal individuals, olfactory function was related to brain atrophy in specific areas and neuropsychological changes in specific domains (i.e., the entorhinal cortex, hippocampus, and frontal and temporal areas) over time (Tian et al., 2023).

These reports suggested that olfactory impairment in older adults was a marker of potential problems with physical and brain dysfunction (Jobin et al., 2023; Yuan et al., 2024). Therefore, it may be assumed that there is a relationship between olfactory impairment and economic loss. However, it remains unclear how economic loss relates to olfactory impairment among community-dwelling older adults. Only one previous study examined associations between olfactory impairment and total healthcare expenditure (Man et al., 2024). That study examined direct healthcare expenditures, which comprised hospitalization and emergency department visit costs over the previous 6 months, along with mental health and outpatient service use. However, a non-significant association was observed (Man et al., 2024). Some previous studies have shown that olfactory impairment in community-dwelling older adults is associated with early, mild gait disturbances and subtle brain atrophy—both of which fall within normal limits and often go unnoticed by the individuals themselves (Kose et al., 2021; Kose et al., 2022). Therefore, it may be assumed that olfactory impairment among community-dwelling older adults is related to mild or moderate health concerns (e.g., internal or orthopedic), rather than serious or severe concerns (e.g., hospitalization or emergency department visits). The present study aimed to extend current knowledge regarding the relationship between olfactory performance and social factors among physically independent, community-dwelling older adults in Japan. Therefore, we examined whether better olfaction was associated with lower healthcare expenditure using both a cross-sectional analysis and a longitudinal pilot study.

## Methods

### Study population

The current study was conducted in Yanai city (Yamaguchi, Japan; this experiment was part of a larger study called the Yanai Study) (Kimuro et al., 2019). Participants in the Yanai Study were 181 individuals (mean age: 70.6 ± 5.6 years; age range: 64–88 years; 59 men and 122 women) recruited in 2015. Individuals were excluded from this study if they had not completed tests for olfaction and healthcare expenditure (n = 1) or if they had suspected Alzheimer’s disease (global cognition test score <13) (Inoue et al., 2009) or suspected depression [Geriatric Depression Scale (GDS) score >6] (Yesavage, 1988; Sugishita et al., 2017) (n = 18). Finally, 162 participants with full data were included in the analyses (mean age: 70.2 ± 5.4 years; range: 64–88 years; 48 men and 114 women). Of these, we followed 71 community-dwelling older adults (mean age 70.0 ± 5.7 years; range: 64–88 years; 26 men and 45 women) in a longitudinal study over a 1.5-year period. We excluded 91 community-dwelling older adults (mean age 70.3 ± 5.1 years; 22 men and 69 women) from this longitudinal analysis because they were enrolled in an exercise intervention program.

For the cross-sectional analyses, the measurement period for olfaction, physical performance, cognitive function, and comorbidities was from May to August 2015. The baseline measurement period of health expenditure was between May 2014 and April 2015. In the longitudinal pilot study, there was no additional measurement period for olfaction, physical performance, cognitive function, and comorbidities beyond the cross-sectional measurement period. All health expenditure indices were additionally measured between May 2015 and October 2016.

This study was conducted in accordance with guidelines laid down in the Declaration of Helsinki, and all procedures involving human participants were approved by the Ethics Committee of Fukuoka University in Japan (approval no. 14-05-01). The purpose, procedures, and risks of the study were explained to each participant. All participants provided written informed consent before participating in the study.

### Olfaction

We examined participants’ ability to identify odors using the Odor Stick Identification Test for Japanese people (OSIT-J, produced by Daiichi Yakuhin Sangyo Co., Ltd.) (Kobayashi et al., 2006; Saito et al., 2006). The tests included 12 odors that consisted of Indian ink, wood, menthol, Japanese cypress [hinoki], perfume, rose, Japanese orange, condensed milk, roasted garlic and curry, cooking gas, and fermented beans/sweaty socks. These odorants are encountered regularly in everyday life in Japan and are considered to be familiar to most individuals in the Japanese population. Each odorant consisted of a solid cream enclosed in a microcapsule shaped like a lipstick.

Participants were instructed to choose the correct odor from four alternatives; however, they could choose “detectable but not recognized” or “no smell detected” if they could not identify the odor as one of the four alternatives (the four-plus alternative method) (Saito et al., 2006). For example, when participants were tested on Japanese orange odor, the response options were as follows: “banana,” “apple,” “peanut,” “Japanese orange,” “detectable but not recognized,” and “no smell detected.” A correct answer for an odor was scored as 1 and an incorrect answer as 0. The overall olfaction score was calculated from the scores for 12 odors based on the number of correct answers.

We divided participants into tertile groups based on the olfaction score (T1: n = 49 [score range 0–7]; T2: n = 44 [score range 8–9]; and T3: n = 69 [score range 10–12]) (Kose et al., 2021; Kose et al., 2022). Olfactory subgroups were defined objectively (i.e., without the researchers’ subjective opinions) and grouped using SPSS automatic grouping based on the observed percentiles in the study population. The olfactory cut-off value used to classify both healthy adults and older adults was 7/8 (Fujio et al., 2012). This value was not used for older adults only. As this study only included independent older adults in the community, we used these data to create tertiles based on the number of participants.

### Healthcare expenditure

All health expenditure data were obtained from official databases maintained by Yanai City Hall staff. In Japan, healthcare data are stored in a format specific to each city, town, and village. These healthcare expenditure data were checked against the paper records for individuals on their medical expense sheets by Yanai City Hall staff. As this work required a lot of effort and time, we needed to consider this burden on staff when requesting data. However, we requested the extraction of as much data as possible, including total healthcare expenditure, outpatient visit costs, and internal medicine and orthopedic expenditure. Finally, we collected healthcare expenditure data for eight indices: total healthcare expenditure, outpatient visits, internal medicine, orthopedics, cerebral surgery, ophthalmology or otology, drug treatment, and admission. Total healthcare expenditure consisted of outpatient visits and drug treatment. Outpatient visits included services related to internal medicine, orthopedics, cerebral surgery, and ophthalmology and/or otology. These indices and hospital admission costs were measured in United States dollars [USD] (1 USD = 144 Japanese yen).

### Cognitive function

Global cognition was tested using a simple test battery for Alzheimer’s disease screening in community-based settings (Inoue et al., 2009). This test consisted of 15 points across four category tasks: an immediate memory test, a temporal orientation test, a three-dimensional visuospatial perception test, and a delayed recall test. Part A of the Trail Making Test (TMT-A) was used to assess executive functions, especially processing speed. In TMT-A, participants were required to draw a line between encircled numbers (1, 2, 3, and 4). Lower scores indicated a better performance (Reitan, 1955).

### Physical performance, physical parameters, and comorbidities

We examined four physical function tests: timed up-and-go (Podsiadlo D, 1991), five-time chair stand, hand grip strength, and one-leg standing with eyes open. Descriptions of these tests have been published by Kimura et al. (2012).

The timed up-and-go test (Podsiadlo D, 1991) used a mat sensor (TKK5804; Takei Scientific Instruments, Niigata, Japan). Participants sat on a standard chair (height: 0.4 m) without armrests, wearing conventional clothes but no footwear. The test performance time was recorded using a stopwatch. Participants were asked to stand up, walk 3 m, turn around, walk back, and sit down as quickly as possible. Lower scores indicated better performance. Two trials were completed, and the better score was recorded.

For the five-time chair stand test, participants sat on a standard chair (height: 0.4 m, without armrests), with their arms folded across the front of their upper body. The time required for participants to stand up and sit down five times was measured. Two trials were completed, and the better score was recorded (Kimura et al., 2012).

Hand grip strength was measured using a Smedley Hand Dynamometer (Grip-D, TKK5101; Takei Scientific Instruments, Niigata, Japan). Participants were instructed to maintain an upright position with their arm completely extended and not touch their body with the hand dynamometer. Participants were encouraged to produce their maximum hand grip power. Two trials were conducted with each hand. The better score of the two trials was recorded (Kimura et al., 2012).

For the one-leg standing with eyes open test, the amount of time that the participant was able to balance on one foot with their eyes open was measured using a stopwatch. For this test, participants were barefoot and placed their hands on their hips. They were instructed not to let their legs touch each other and not to move the foot that was standing on the floor (Kimura et al., 2012).

Height and weight were measured using a standard stadiometer and weighing machine. Body mass index was calculated as weight (kg)/height (m)^2^. Depression symptoms were measured using the GDS (Yesavage, 1988; Sugishita et al., 2017). Data on comorbidities (smoking, stroke, cardiovascular disease, diabetes mellitus, hypertension, hyperlipidemia, cancer, orthopedic conditions, and disorders the thyroid, nervous system, and lung and immune system) and smoking history were self-reported.

### Statistical analyses

All healthcare expenditure indices were inverse log-transformed because the variables were skewed.

Analysis of variance (ANOVA) with Bonferroni *post hoc* pairwise comparisons was performed to compare olfaction, physical parameters, and physical performance among the olfaction tertile groups. Analysis of covariance (ANCOVA) with Bonferroni *post hoc* pairwise comparisons was performed to compare healthcare expenditure indices among the tertile olfaction groups. Model A was adjusted for age, sex, GDS, BMI and comorbidities (smoking, stroke, cardiovascular disease, diabetes mellitus, hypertension, hyperlipidemia, cancer, orthopedic conditions, and issues relating to thyroid, nervous system, and lung or immune system). Model B includes all variables from Model A, global cognitive function, TMT-A, timed up-and-go, five-time chair stand, handgrip strength and one leg standing with eyes open. Covariates were determined based on previous studies (Pellegrino et al., 2016; Peng et al., 2019; Yuan et al., 2024). The risk factors for olfactory dysfunction examined were age, sex, obesity (BMI), depression (GDS), neurological disease (cognitive function), other comorbidities, and mobility impairment (physical performance).

Chi-square tests were used to compare physical parameters and comorbidities among the tertile olfaction groups. Logistic regression analysis was performed to determine whether orthopedic expenditure could predict overall olfaction. We set the minimal cut-off score as 4, which reflected severe hyposmia as assessed using the OSIT-J (Baba et al., 2012). We aimed to show that 1) whether it is necessary to set an alternative cut-off score for olfactory dysfunction and 2) that in further research, OSIT-J point-by-point scores may hold meaningful interpretative value. Therefore, we examined the optimal olfaction cut-off score using a sliding range from 4 to 11 (the maximal score was 12).

We divided healthcare expenditure indices into three periods: 0.5 years (May 2015–October 2015), 1.0 years (May 2015–April 2016), and 1.5 years (May 2015–October 2016). Moreover, we divided participants into two olfaction score groups based on the values used in the cross-sectional study [≥8 or ≤7 (7/8 score)].

We used ANCOVA with the Bonferroni *post hoc* test for the analysis of main effects to determine whether olfaction ability was associated with healthcare expenditure over the prospective 1.5 years of follow-up. The independent covariates used in models A and B were the same covariates used in the cross-sectional study. The level of statistical significance was set at *p* < 0.05. All analyses were performed using SPSS v26 for Windows.

## Results

1. Physical parameters, comorbidities, cognitive function, and physical performance based on the olfaction tertile groups (Table 1; Supplementary Table S1)

**TABLE 1:** Characteristics of participants by olfaction tertile groups (cross-sectional analysis).

Variable	All (n = 162)	Olfaction tertile group		
		T1 (n = 49)	T2 (n = 44)	T3 (n = 69)
Olfaction, mean ± SD				
Overall olfaction (12 odors), score	8.4 ± 2.9	4.7 ± 2.1	8.5 ± 0.5	10.9 ± 0.8^∗^†
Overall olfaction (12 odors), score range	0 to 12	0 to 7	8 to 9	10 to 12
Physical parameters, mean ± SD				
Sex, M/F (% female)	48/114 (70)	19/30 (61)	12/32 (73)	17/52 (75)
Age, yrs	70.2 ± 5.4	73.6 ± 5.5	68.8 ± 4.7	68.7 ± 4.6^∗^†
Height, cm	155.3 ± 8.2	156.0 ± 8.9	155.0 ± 9.0	154.9 ± 7.0
Weight, kg	55.0 ± 9.5	55.7 ± 10.4	55.3 ± 9.3	54.4 ± 9.2
BMI, kg/m^2^	22.7 ± 2.9	22.7 ± 2.7	23.0 ± 3.1	22.6 ± 3.0
GDS, score	1.8 ± 1.8	1.9 ± 1.8	2.0 ± 1.9	1.6 ± 1.7

Note: M, male; F, female; BMI, body mass index; GDS, Geriatric Depression Scale; SD, standard deviation.

^*^ *p* < 0.05 in one-way analysis of variance; † *p* < 0.05 in linear trend.

For sex and smoking, *p*-values for olfaction tertile groups were calculated using chi-square tests.

Comorbidities are shown in Supplementary Table S1.

Age and performance on the Trail Making Test, timed up-and-go, and five-time chair stand tests were slightly lower among those with better olfaction than those with worse olfaction (*p* < 0.001, *p* = 0.002, and *p* < 0.001, respectively; *p* = 0.005 for linear trend). Performance on the one-leg standing with eyes open test was better in those with better olfaction than those with worse olfaction (*p* = 0.027 for linear trend). Statistically significant differences were observed among tertiles for the other examined variables. Participants in tertiles T2 and T3 were approximately 4 years younger than those in T1 (p < 0.001 for both). Performance on the Trail Making Test in tertile T3 was approximately 16 s faster than that in T1 (p = 0.006). Performance on the timed up-and-go test in tertile T3 was approximately 0.7 s faster than that in T1 (p < 0.001). Performance on the five-time chair stand test in tertile T3 was approximately 0.9 s faster than that in T1 (p = 0.014). Performance on the timed up-and-go test in tertile T3 was approximately 0.7 s faster than that in T1 (p < 0.001). Finally, performance on the one-leg standing with eyes open test in tertile T2 was approximately 13 s longer than that in T1 (p = 0.022).

Chi-square tests were used to calculate *p*-values for several variables: sex (*p* = 0.234), smoking status (*p* = 0.882), stroke (*p* = 0.278), cardiovascular disease stroke (*p* = 0.329), diabetes mellitus (*p* = 0.112), hypertension (*p* = 0.168), hyperlipidemia (*p* = 0.272), cancer (*p* = 0.347), orthopedic conditions and thyroid disorders (*p* = 0.839), nervous system disorders (*p* = 0.255), and lung and immune system disorders (*p* = 0.534). The results for sex are shown in Table 1, and those for the other variables are presented in Supplementary Table S1.

2. Healthcare expenditure based on the olfaction tertile groups (Table 2)

**TABLE 2:** Healthcare expenditure by olfaction tertile groups (cross-sectional analysis).

Variable	Olfaction tertile group			*p*-value for linear trend		
	T1, n = 49	T2, n = 44	T3, n = 69	Crude	Model A	Model B
	Score range: 0–7	Score range: 8–9	Score range: 10–12			
Total healthcare expenditure	1,572 ± 3,080	1,391 ± 2,188	1,138 ± 2,559	0.646	0.256	0.522
Outpatient visits	637 ± 732	686 ± 945	481 ± 541	0.595	0.229	0.508
Internal medicine	374 ± 467	240 ± 314	301 ± 373	0.431	0.169	0.295
Orthopedics	**89** ± **191**	**51** ± **143**	**52** ± **157**	**0.048**	**0.030**	**0.034**
Cerebral surgery	64 ± 198	115 ± 346	32 ± 107	0.830	0.698	0.671
Ophthalmology and/or otology	110 ± 243	279 ± 765	95 ± 233	0.489	0.804	0.714
Drug	444 ± 668	235 ± 293	581 ± 2,291	0.789	0.480	0.740
Admission	491 ± 2,144	470 ± 1,444	76 ± 415	0.390	0.595	0.666

Note: *p* < 0.05 indicated in bold. Mean ± SD.

All healthcare expenditure indices were inverse log-transformed for statistical analysis because these variables were skewed.

The table shows scores, not log-transformed data (United States dollar [USD], 1 USD = 144 Japanese yen).

*p*-values for olfaction tertile groups were calculated using analysis of covariance.

Model A adjusted for age, sex, body mass index, geriatric depression scale, and comorbidities (smoking, stroke, cardiovascular disease, diabetes mellitus, hypertension, hyperlipidemia, cancer, orthopedic conditions, and issues relating to thyroid, nervous system, and lung or immune system).

Model B adjusted for Model A variables, cognitive function (global cognitive and Trail Making Test-A), and physical performance (timed up-and-go, five-time chair stand, hand grip strength, and one-leg standing with eyes open).

Model A: T1, n = 49; T2, n = 42; and T3, n = 68; Model B: T1, n = 42; T2, n = 36; and T3, n = 63.

No associations were found using ANOVA and ANCOVA with Bonferroni *post hoc* pairwise comparisons when comparing all orthopedic expenditure indices among the tertile olfaction groups.

Better olfaction was associated with lower orthopedic expenditure in the crude model and all covariate models (crude: *f* = 2.19, *p* for ANOVA = 0.115, and *p* for linear trend = 0.048; Model A: *f* = 2.68, *p* for ANCOVA = 0.071, and *p* for linear trend = 0.030; Model B: *f* = 2.30, *p* for ANCOVA = 0.105, and *p* for linear trend = 0.034). No associations were found using ANOVA and ANCOVA with Bonferroni *post hoc* pairwise comparisons when comparing all orthopedic expenditure indices among the tertile olfaction groups. Other types of healthcare expenditure were not associated with olfaction.

3. Logistic regression analysis for predicting olfaction (empirically derived thresholds) from orthopedic expenditure (Table 3)

**TABLE 3:** Logistic regression analysis for predicting olfaction (empirically derived thresholds) based on orthopedic expenditure.

Variable	Model A (n = 159)			Model B (n = 141)		
	n	OR	95% CI	n	OR	95% CI
11/12 score	141/21	1.00	0.726–1.37	125/16	1.05	0.735–1.49
10/11 score	119/40	0.988	0.796–1.23	105/36	1.03	0.804–1.31
9/10 score	91/68	0.872	0.716–1.06	78/63	0.793	0.623–1.01
8/9 score	72/87	0.832	0.689–1.01	61/80	**0.760**	**0.601**–**0.962** ^∗^
7/8 score	49/110	**0.770**	**0.622**–**0.955^∗^**	42/99	**0.780**	**0.612**–**0.994** ^∗^
6/7 score	38/121	0.932	0.750–1.16	34/107	0.905	0.707–1.16
5/6 score	28/131	0.938	0.739–1.19	26/115	0.899	0.680–1.19
4/5 score	16/143	0.842	0.627–1.13	15/126	0.849	0.575–1.26

Note: *p* < 0.05 indicated in bold. OR, odds ratio; CI, confidence interval.

All healthcare expenditure indices were inverse log-transformed for statistical analysis because these variables were skewed.

*p*-values for olfaction tertile groups were calculated using logistic regression analysis.

^*^ *p* < 0.05 in logistic regression analysis.

The median olfaction score was 9.

Poorer olfaction cut-off scores = 0; better olfaction cut-off scores = 1.

Model A adjusted for age, sex, body mass index, Geriatric Depression Scale, and comorbidities (smoking, stroke, cardiovascular disease, diabetes mellitus, hypertension, hyperlipidemia, cancer, orthopedic conditions, and issues relating to thyroid, nervous system, and lung or immune system).

Model B adjusted for Model A variables, cognitive function (global cognitive and Trail Making Test-A), and physical performance (timed up-and-go, five-time chair stand, hand grip strength and one-leg standing with eyes open).

Model A: T1, n = 49; T2, n = 42; and T3, n = 68; Model B: T1, n = 42; T2, n = 36; and T3, n = 63.

Based on an olfaction cut-off score of 7/8 (reference: ≤7 group), a better olfaction score (≥8) was associated with lower orthopedic expenditure in Model A [odds ratio (OR): 0.770; 95% confidence interval (CI): 0.622–0.955; *p* = 0.017] and Model B (OR: 0.780; 95% CI: 0.612–0.994; *p* = 0.045).

Based on an olfaction cut-off score of 8/9 (reference: ≤8 group), a better olfaction score (≥9) was associated with lower orthopedic expenditure in Model B (OR: 0.760; 95% CI: 0.601–0.962; *p* = 0.022).

To assess multicollinearity, we calculated the variance inflation factor (VIF) for all variables, which ranged from 1.1 to 4.3.

4. Healthcare expenditure in the longitudinal analysis (prospective 1.5 years) using olfaction cut-off scores of 7/8 (n = 71) (Table 4)

**TABLE 4:** Healthcare expenditure by olfaction cut-off score (7/8) groups (longitudinal pilot study).

Variable	Health expenditure for each year			*p*-value for time × group interaction	
	Following 0.5 years	Following 1.0 years	Following 1.5 years	Model A	Model B^a^
Total healthcare expenditure					
≤7	**723** ± **1,532**	**3,164** ± **9,004**	**4,057** ± **9,404**	**0.037**	**0.021**
≥8	**792** ± **2,661**	**1,375** ± **3,385**	**2,073** ± **4,140**		
Outpatient visits					
≤7	**297** ± **360**	**672** ± **750**	**1,077** ± **1,230**	**0.022**	**0.016**
≥8	**293** ± **326**	**611** ± **791**	**874** ± **1,115**		
Internal medicine					
≤7	**185** ± **267**	**437** ± **550**	**752** ± **1,003**	**0.001**	**0.005**
≥8	**169** ± **220**	**329** ± **480**	**463** ± **685**		
Orthopedics					
≤7	72 ± 180	171 ± 389	205 ± 503	0.689	0.836
≥8	26 ± 64	59 ± 132	82 ± 184		
Cerebral surgery					
≤7	16 ± 55	16 ± 55	36 ± 96	0.667	0.478
≥8	30 ± 94	52 ± 153	72 ± 212		
Ophthalmology and/or otology					
≤7	24 ± 66	49 ± 123	84 ± 207	0.303	0.670
≥8	69 ± 99	171 ± 293	257 ± 396		
Drug					
≤7	204 ± 373	2,117 ± 8,481	2,350 ± 8,543	0.267	0.362
≥8	122 ± 176	269 ± 392	396 ± 593		
Admission					
≤7	222 ± 1,111	375 ± 1,322	630 ± 1,782	0.865	0.913
≥8	377 ± 2,405	495 ± 2,517	803 ± 2,852		

Note: *p* < 0.05 indicated in bold. Mean ± standard deviation (SD).

Real healthcare expenditure [United States dollar (USD), 1 USD = 144 Japanese yen] is shown; however, all healthcare expenditure indices were inverse log-transformed in all models because these variables were skewed.

*p*-values for olfaction cut-off (7/8) groups were calculated using two-way analysis of covariance.

Model A adjusted for age, sex, body mass index, Geriatric Depression Scale, and comorbidities (smoking, stroke, cardiovascular disease, diabetes mellitus, hypertension, hyperlipidemia, cancer, orthopedic conditions, or issues relating to thyroid, nervous system, and lung or immune system).

Model B adjusted for Model A variables, cognitive function (global cognitive and Trail Making Test-A), and physical performance (timed up-and-go, five-time chair stand, and one-leg standing with eyes open).

^a^ Model B: ≤7 group: n = 22; ≥8 group: n = 40.

Participants were divided into two groups using an olfaction cut-off score of 7/8. A better olfaction score (≥8) was associated with lower total healthcare expenditure, outpatient visit costs, and internal medicine expenditure compared with a poorer olfaction score (≤7), as shown in the two-way ANCOVA adjusted for covariates in Model A (group × time interaction: *f* = 3.40, *p* = 0.037; *f* = 3.93, *p* = 0.022; and *f* = 7.65, *p* = 0.001) and Model B (group × time interaction: *f* = 4.05, *p* = 0.021; *f* = 4.34, *p* = 0.016; and *f* = 6.20, *p* = 0.005).

## Discussion

We examined whether better olfaction was associated with lower healthcare expenditure using both a cross-sectional analysis and a longitudinal pilot study. We observed two main findings. First, better olfactory identification was associated with lower orthopedic expenditure in the cross-sectional analysis. Second, better olfaction identification was associated with reduced total healthcare, outpatient visit costs, and internal medicine expenditure in the longitudinal pilot study over 1.5 years.

To our knowledge, few previous studies examined the association between olfaction and healthcare expenditure among older adults (Man et al., 2024). A previous study assessed direct total healthcare expenditure using hospitalization and emergency department visit costs over the past 6 months, along with mental health and outpatient service use over the past 3 months, among older Asian adults (N = 2,643) but reported a non-significant association (Man et al., 2024). We speculated on reasons for the differences between that study and our study. First, our healthcare data were collected over longer periods (1 year in the cross-sectional analysis and 1.5 years in the longitudinal pilot study) than the previous study. Second, we focused on mild or moderate health concerns (e.g., internal and orthopedic), rather than serious or severe concerns (e.g., hospitalization and emergency department visits). We speculated that the associations between olfaction ability and healthcare expenditures were blind. These findings may support an association between olfaction ability and mild or moderate health concerns among physically independent, community-dwelling older adults.

Our cross-sectional analysis showed that there was a relationship between olfactory performance and orthopedic expenditure, but no difference was observed in the small amount of orthopedic expenditure (approximately 30–40 USD/person/year; olfaction tertile group 1: T1 = 89 ± 191 USD/year/person vs. T3 = 52 ± 157 USD/person/year, as shown in Table 2). In Japan, pain relief patches are commonly used as a symptomatic treatment for vague symptoms. The cost of pain relief patches prescribed by an orthopedic surgeon is lower than that of over-the-counter medications, and older adults often visit an orthopedic surgeon when they have body pain. The cost of pain relief patches (Loxonin Tape 50 mg, 7 sheets/pack) was approximately 1 USD. Therefore, orthopedic expenditure tends to be lower than other types of medical expenses (e.g., internal medicine). For example, the difference in the present findings may suggest that individuals with worse olfaction were prescribed approximately 2–3 packs of pain relief patches/month/person more than those with better olfaction. Specifically, we speculated that individuals with poorer olfaction visited an orthopedic approximately once/month/person more than those with better olfaction. These findings suggested an association between olfactory impairment and regular medical visits for mild–moderate health concerns.

In our longitudinal analysis, better overall olfaction at baseline was associated with lower total healthcare, outpatient visit, and internal medicine expenditure over 1.5 years (Table 4). Previous studies conducted among community-dwelling older adults showed that poorer olfactory function could serve as a predictor of mortality (Pinto et al., 2014) and dementia (Knight et al., 2023). Moreover, previous reports on the association between olfaction and internal factors suggested that impaired olfaction may gradually degrade one’s food choices, which adversely affect dietary quality and nutrition (Gopinath et al., 2016; Roxbury et al., 2022). Poor olfactory function was also associated with reduced kidney function (Wang et al., 2022), and olfaction test results were associated with a long-term risk of incident coronary heart disease (Chamberlin et al., 2024). These reports suggested that poorer olfaction influenced internal physiological factors and contribute to undesirable health behaviors over time. Therefore, these previous findings may support our results. We also showed that a small difference in healthcare expenditure in the cross-sectional analysis could lead to a significant increase in expenditure over time, as demonstrated in the longitudinal pilot study. Therefore, even if a difference is small in a cross-sectional analysis, this difference should not be ignored in the long term. However, our longitudinal study enrolled a relatively small number of individuals (n = 71). Further studies should use longer follow-up periods and include larger sample sizes to examine healthcare expenditure in more detail.

The present study covered the information for 2015–2016. Starting on 1 October 2022, Japan’s Ministry of Health, Labor, and Welfare increased the out-of-pocket medical expense burden from 10% to 20% for people aged 75 years or older who had an income above a certain level, excluding those whose income was equivalent to that of working people. The purpose of this change was to establish a social security system that offer a sense of security for all generations while minimizing the increased burden on the working generation as they approach the age group of 75 years and older. However, it may be difficult for people over 75 years to visit hospitals as frequently as they did previously. If this is the case, the number of older people who do not visit hospitals even when they have minor pain may increase, which may lead to reduced medical costs. Therefore, the relationship between olfactory ability and medical costs may not be as strong now as it was in 2015–2016, but the importance of olfactory ability in identifying older people with declining health may increase. We based the olfactory cut-off score on findings from previous research. A previous study that used the Brief Smell Identification Test to categorize olfaction (anosmia: score ≤6, hyposmia: a score of 7–8, moderate function: a score of 9–10, and good function: a score of 10–11) found that poorer olfaction was associated with reduced kidney function (Wang et al., 2022). Another study used the 12-item Brief Smell Identification Test to define olfaction (poor: score ≤8, moderate: a score of 9–10, and good: a score of 11–12) and reported an association with a long-term risk of incident coronary heart disease (Chamberlin et al., 2024). Among Japanese people (mean age 57 years; range 18–77 years), olfaction scores ≥8 on the Open Essence test are considered normal (Fujio et al., 2012). Another study showed that olfactory impairment (an Open Essence score ≤7) was closely associated with sarcopenia and frailty in community-dwelling older adults (Harita et al., 2019). In the context of Parkinson’s disease, patients with severe hyposmia (score ≤4) had an 18.7-fold increased risk for dementia for each 1 standard deviation (2.8) decrease in the OSIT-J score (Baba et al., 2012).

Previous studies conducted among community-dwelling older adults reported that olfactory dysfunction was associated with poor physical performance (Tian et al., 2017; Kose et al., 2022; Yuan et al., 2024), cognitive function (Delgado-Lima et al., 2023), and brain structure (Kose et al., 2021; Bothwell et al., 2023; Tian et al., 2023). Findings from a previous report (Kose et al., 2022) based on the same cohort suggested that associations between olfaction and physical performance indicated early aging, with poorer olfaction being associated with a mild decrease in physical performance. Another study showed that, among community-dwelling Japanese older adults defined as having olfactory impairment (assessed using an Open Essence score ≤7, with the full 12 cards and odor selections described by the OSIT-J), olfactory impairment was closely associated with sarcopenia and frailty (Harita et al., 2019). These reports and the findings of our cross-sectional analysis may help support the idea that worse olfaction may indicate potential physical problems.

Previous findings linked declines in sensory abilities to health problems in older adults. For example, a previous review showed that hearing impairment was associated with numerous health issues, including accelerated cognitive decline, depression, increased risk for dementia, poor balance, falls, hospitalization, and early mortality (Davis et al., 2016). The risk for all-cause mortality was higher in people with vision impairment than in those with normal vision or mild vision impairment, and the magnitude of this effect increased with more severe vision impairment (Ehrlich et al., 2021). Interestingly, another review (Bathini et al., 2024) showed that specific peripheral sensory systems, such as the nose and olfactory bulb, are directly vulnerable to external agents, including microbes. The sensory organ for hearing (the ear) is equipped with a barrier known as the blood–labyrinthine barrier but also has resident immune cells and can be susceptible to infections that may affect the central nervous system (Hirose et al., 2014). The eye, which is a unique sensory organ with its own immune system, has direct contact with the external environment, with the cornea and conjunctiva acting as barriers. However, certain infections can impair the eye and have potential to impact the central nervous system under specific circumstances (Bathini et al., 2024). Although the brain is protected by the blood–brain barrier, microbes may enter through the bloodstream, neural pathways, intranasal route, sinuses, the oral cavity, and the gut–brain axis. These suggest that accelerated impairment in sensory abilities can reflect a shift from healthy to pathological aging, including the development of Alzheimer’s disease and other neurological disorders, which is consistent with the “gateway hypothesis” (Bathini et al., 2024). If microbial mechanisms of sensory dysfunctions are identified as causally related to neurodegenerative processes, accessible and inexpensive smell, visual, or hearing testing could represent the first line of routine diagnostics (Bathini et al., 2024).

The pathway from olfactory decline to increased healthcare costs remains unclear. However, as olfactory dysfunction can reflect the decline of physical and brain functions and health level, we speculated pathways from olfactory decline to increased healthcare costs as follows: 1) poorer olfaction may indicate potential physical problems, and individuals with poorer olfaction may visit an orthopedic physician more often than individuals with better olfaction. 2) Poorer olfaction may indicate potential physical and cognitive problems (including brain structure changes) and potential risks for obesity, kidney disease, and coronary heart disease, all of which are associated with dietary quality and nutrition. Therefore, regular medical visits (e.g., internal medicine and neurology) are needed in the long term. 3) In the long term, individuals with poorer olfaction may shift from healthy to pathological aging, including the development of Alzheimer’s disease and other neurological disorders. Therefore, they may need regular medical visits because of severe health concerns.

The present study had several limitations. First, it remains unclear whether the characteristics of participants in our study were similar to those of the general population or whether the sample size and follow-up period in the longitudinal pilot study were sufficient to provide adequate statistical power. Second, the sex distribution of our sample was imbalanced, with a higher proportion of female participants. Given that olfactory performance is generally poorer in men than in women (Doty et al., 1985), this may have resulted in overall olfactory scores being higher than expected in a more gender-balanced population. Therefore, our findings may not be fully generalizable. However, to minimize bias in group comparisons, we confirmed that the sex distribution did not significantly differ among the tertile groups used in the cross-sectional analysis. Furthermore, sex was included as a covariate in all statistical models to control for its potential confounding effect. Third, associations between olfaction and healthcare expenditure among non-Japanese people remain unclear. Finally, participants with suspected Alzheimer’s disease and depression were excluded from our analyses. However, we were unable to exclude participants with suspected mild cognitive impairment at risk for Alzheimer’s disease, early Parkinson’s disease, or dementia with Lewy bodies. Several previous studies reported that Parkinson’s disease (Takeda et al., 2014), Alzheimer’s disease (Mesholam et al., 1998), and dementia with Lewy bodies (Ross et al., 2006) were associated with hyposmia. Furthermore, it remains unclear whether the present findings were affected by other diseases or disorders that were not measured. Further studies should examine additional screening tests in more depth and use self-report questions covering various other diseases or disorders.

## Conclusion

The study found that improved olfaction was associated with decreased orthopedic expenditure in 2015, along with reduced total healthcare, outpatient visit, and internal medicine expenditures from 2015 to 2016. These findings suggest that raising awareness about olfactory dysfunction among physically independent Japanese older adults may be beneficial. Olfaction testing may be easily integrated into health screening protocols for aging populations, along with vision and hearing tests, and could be used to anticipate increasing healthcare costs.

## Data Availability

The raw data supporting the conclusions of this article will be made available by the authors, without undue reservation.

## Abbreviations

OSIT-J, the Odor Stick Identification Test for Japanese people; SD, standard deviation; GDS, Geriatric Depression Scale; OR, odds ratio; CI, confidence interval; TMT, Trail Making Test; ANOVA, analysis of variance; ANCOVA, analysis of covariance.
