# Supplementary material for: Better olfaction was associated with lower healthcare expenditure among physically independent Japanese community-dwelling older adults: the Yanai study
Source: Front Aging. 2025 Aug 7;6:1592838. doi: 10.3389/fragi.2025.1592838 (PMC12367730; doi:10.3389/fragi.2025.1592838)
Supplement: Supplementary file 1 [file Presentation1.pptx]

## Slide 1
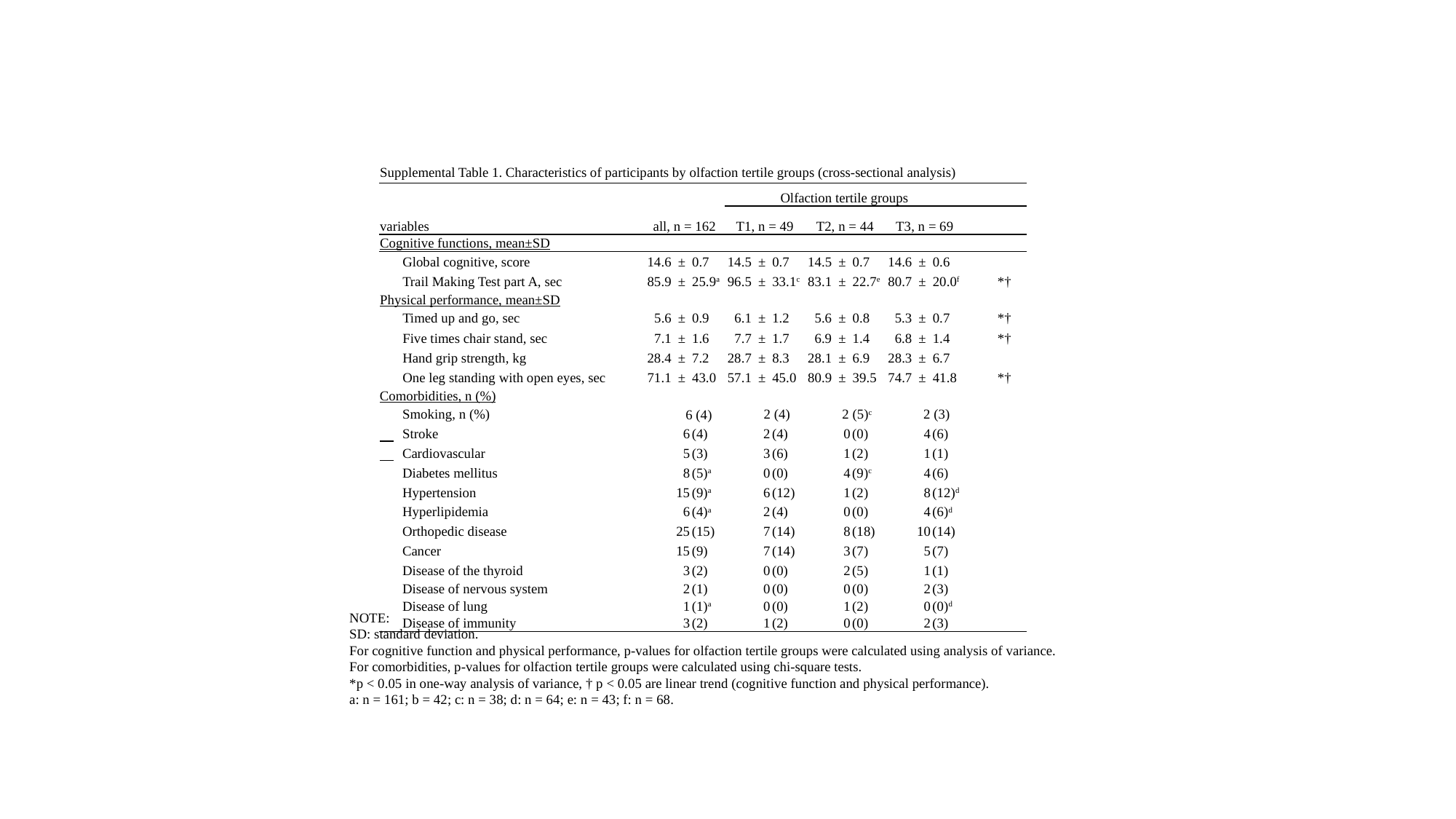

| Supplemental Table 1. Characteristics of participants by olfaction tertile groups (cross-sectional analysis) | | | | | | | | | | | | | | | |
| --- | --- | --- | --- | --- | --- | --- | --- | --- | --- | --- | --- | --- | --- | --- | --- |
| variables | | all, n = 162 | | | Olfaction tertile groups | | | | | | | | | | |
| | | | | | T1, n = 49 | | | T2, n = 44 | | | T3, n = 69 | | | | |
| Cognitive functions, mean±SD | | | | | | | | | | | | | | | |
| | Global cognitive, score | 14.6 | ± | 0.7 | 14.5 | ± | 0.7 | 14.5 | ± | 0.7 | 14.6 | ± | 0.6 | | |
| | Trail Making Test part A, sec | 85.9 | ± | 25.9a | 96.5 | ± | 33.1c | 83.1 | ± | 22.7e | 80.7 | ± | 20.0f | | \*† |
| Physical performance, mean±SD | | | | | | | | | | | | | | | |
| | Timed up and go, sec | 5.6 | ± | 0.9 | 6.1 | ± | 1.2 | 5.6 | ± | 0.8 | 5.3 | ± | 0.7 | | \*† |
| | Five times chair stand, sec | 7.1 | ± | 1.6 | 7.7 | ± | 1.7 | 6.9 | ± | 1.4 | 6.8 | ± | 1.4 | | \*† |
| | Hand grip strength, kg | 28.4 | ± | 7.2 | 28.7 | ± | 8.3 | 28.1 | ± | 6.9 | 28.3 | ± | 6.7 | | |
| | One leg standing with open eyes, sec | 71.1 | ± | 43.0 | 57.1 | ± | 45.0 | 80.9 | ± | 39.5 | 74.7 | ± | 41.8 | | \*† |
| Comorbidities, n (%) | | | | | | | | | | | | | | | |
| | Smoking, n (%) | 6 (4) | | | 2 (4) | | | 2 (5)c | | | 2 (3) | | | | |
| | Stroke | | 6 | (4) | | 2 | (4) | | 0 | (0) | | 4 | (6) | | |
| | Cardiovascular | | 5 | (3) | | 3 | (6) | | 1 | (2) | | 1 | (1) | | |
| | Diabetes mellitus | | 8 | (5)a | | 0 | (0) | | 4 | (9)c | | 4 | (6) | | |
| | Hypertension | | 15 | (9)a | | 6 | (12) | | 1 | (2) | | 8 | (12)d | | |
| | Hyperlipidemia | | 6 | (4)a | | 2 | (4) | | 0 | (0) | | 4 | (6)d | | |
| | Orthopedic disease | | 25 | (15) | | 7 | (14) | | 8 | (18) | | 10 | (14) | | |
| | Cancer | | 15 | (9) | | 7 | (14) | | 3 | (7) | | 5 | (7) | | |
| | Disease of the thyroid | | 3 | (2) | | 0 | (0) | | 2 | (5) | | 1 | (1) | | |
| | Disease of nervous system | | 2 | (1) | | 0 | (0) | | 0 | (0) | | 2 | (3) | | |
| | Disease of lung | | 1 | (1)a | | 0 | (0) | | 1 | (2) | | 0 | (0)d | | |
| | Disease of immunity | | 3 | (2) | | 1 | (2) | | 0 | (0) | | 2 | (3) | | |
NOTE:
SD: standard deviation.
For cognitive function and physical performance, p-values for olfaction tertile groups were calculated using analysis of variance.
For comorbidities, p-values for olfaction tertile groups were calculated using chi-square tests.
*p < 0.05 in one-way analysis of variance, † p < 0.05 are linear trend (cognitive function and physical performance).
a: n = 161; b = 42; c: n = 38; d: n = 64; e: n = 43; f: n = 68.
